# The Treatment of Deep-seated Abscesses without External Incision

**Published:** 1885-09

**Authors:** John S. Marshall

**Affiliations:** Chicago


### The Treament of Deep-seated Abscesses associated with the Teeth without External Incision.

BY JOHN S. MARSHALL, M.D., CHICAGO.

*Mr. President and Gentlemen of the Society:*

The subject which I have the pleasure of presenting to you to-day is one in which I have taken great interest for a considerable time, and have been hoping that the day would come when some means would be suggested whereby its attendant dangers might be lessened and the need for the use of the knife in most cases done away with.

By *deepseated* abscesses I desire that you will understand me as referring to those cases of alveolar abscesses which have extended beyond the ordinary limits, and have involved more or less extensively the structure of the jaw with a tendency to necrosis, or have penetrated the antrum of Highmore, or have escaped from the neighborhood of the maxilla, and have burrowed downward between the muscles of the neck, as frequently occurs in abscesses associated with the inferior teeth.

Ordinarily, the diagnosis of these cases in not difficult, but occasionally the cause of the disease has proved troublesome to find.

Abscesses discharging into the antrum or the nasal fossa and producing offensive discharge have been diagnosed as chronic catarrh. One case occurring in the practice of Dr. Edward Maynard, of Washington, D. C., caused by an unerupted inferior wisdom tooth, and discharging into the larynx, setting up irritative cough with expectoration of pus and mucous, was previously diagnosed by the physicians to be acute bronchitis; others discharging at some point upon the side of the neck, have been set down as abscesses originating in the cervical glands, the result of scrofula.

That such abscesses often prove to be serious affections, endangering the health, and sometimes even the life of the individual, are well established facts.

I propose, however, in this short paper to confine my remarks to the more common, and from their location, the more dangerous class of these cases, viz: those originating from disease of the inferior teeth.

The tendency of the suppurative products in these cases is downwards through the external wall of the alveolar process, and to a point at the lower margin of the jaw; but it also happens—especially with the molar teeth—that instead of pointing at this location it opens through the internal wall of the alveolar process and burrows downwards between the muscles of the neck, and may discharge into the throat, or through the external tissues at various points, from the submaxillary triangle to the superior border of the clavicle.

Any suggestions, therefore, in regard to the treatment of these cases which will tend to cut short the suppurative process, lessen the dangers to health and life, avoid the necessity of operating with the scalpel in a location requiring such delicate dissections and fraught with so much risk to the patient, and prevent the unsightly and oftentimes unsightly scars which follow the external opening of these abscesses, will, I think, be of interest.

The treatment generally adopted in cases of alveolar abscesses, is the removal of the cause by the extraction of the offending tooth, and then usually trusting to nature to complete the cure.

In extreme cases of this deep seated variety an incision is made through the external tissue at the lowest point of the abscess for the purpose of drainage.

In those cases, however, where the pus has burrowed deeply into the tissues of the neck it is quite likely that more than one pocket will be formed, consequently the treatment by incision becomes complicated, and sometimes from the dangers of an extended operation in the superior or inferior carotid triangles would be precluded altogether.

The surgeon under such circumstances has had no alternative but to wait, trusting that the abscess would find an opening for itself at less risk before the patient should die of pyæmia.

The treatment which is suggested comes to our relief in the emergency, and from past experience with it, I am prepared to say, at least, that the duration of these cases can be materially shortened and many of them speedily cured without resort to any other operative procedure than the extraction of the diseased tooth and the injection into the sac of peroxide of hydrogen.

With the introduction to the profession of the peroxide of hydrogen, a valuable antiseptic and germicide has been placed in our hands. Ophthalmologists and aurists have found it very useful in the treatment of diseases of the eye and ear with purulent and muco-purulent discharges, and dentists have been signally successful with it in removing the putrid contents of the pulp chamber in devitalized teeth and in treating the ordinary alveolar abscesses and pyorrhea alveolaris.

By injecting an abscess of this deepseated variety with $H_2 O_2$ introduced through the alveolus of the extracted tooth, the purulent contents can be thoroughly evacuated.

This oxygen is set free on coming in contact with the products of decomposition, distends the cavity and forces out the pus through the alveolus by mechanical pressure.

Two or three injections of ʒss. to ʒj., according to the extent of the abscess, may be required to completely remove the purulent matter, and if given opportunity it will search out and purify every hidden receptacle.

I have had several opportunities since the introduction of $H_2 O_2$ to the notice of dental surgeons by Dr. Walter Coffin of England, at the London International Medical Congress in 1881—and to whom this credit is due—to test its efficiency in this class of cases, and in extensive periosteal inflammation of the jaws.

In one case, a Mercy Hospital patient, Mary N., Irish, aged twenty-four years, suffering with a deep-seated abscess associated with the right inferior wisdom tooth, and which had been progressing for several weeks, and confining the patient to her bed for twenty-six days, with pulse ranging from 100 to 116, and temperature from 101 to 104.8, was speedily relieved by extracting the tooth, and evacuating the pus.

The abscess extending down the neck four inches, below the margin of the gums, as was ascertained by the probe, the pulse and temperature dropped from P. 104. T. 100.8 on the previous day to P. 96. T. 103, two hours after operation. $H_2 O_2$ was ordered to be injected into the abscess ʒss. every four hours; this was followed by a decrease in the temperature of one degree each day for three days.

The patient then refused to submit to the further use of remedy at that time as the evolutions of the gas, by distending the sac, caused pain. This was immediately followed by an increase in the pulse rate, and an elevation in the temperature.

On the fourth day afterwards they had reached P. 101, T. 104.

$H_2 O_2$ was again used as before, and the pulse and temperature again rapidly fell, but through the obstinacy of the patient the treatment could not be carried out with any degree of satisfaction, still the fact was established that the peroxide of hydrogen antisepticized the pus and evacuated the sac as indicated in the rapid improvement in the symptoms.

Another case was a little girl, aged eleven years, with an abscess originating from the right inferior first molar and extending into the tissues of the neck, and tenderness, but with no acute pain.

The swelling of the parts had followed an attack of severe pain in the tooth and jaw, from which she had suffered three weeks previously. For a week the jaws had been closed, and the only food taken each day was a little milk. The child had been confined to the bed for a part of the time and when presented for treatment looked decidedly ill.

The tooth was extracted under ether and the pus cavity found to extend downwards three inches below the margin of the gum. Very little purulent matter followed the extraction of the tooth, but on injecting the pocket with $H_2$ $O_2$ large quantities were evacuated.

The injections of $H_2$ $O_2$ were continued once *per diem* for six days when the patient was discharged cured; all discharge having ceased, and the swelling nearly disappeared.

Under the ordinary treatment I should have expected this case to have continued for a much longer period, and perhaps finally to have seen it point at some location lower down the neck.

Other cases, very much like this one in character, might be mentioned in proof of the value of this remedy, but I desist for fear of wearying you.

One more however might be related with profit. This was a case of a lad nine years of age who had received a traumatic injury of the inferior jaw, by a fall from his bicycle and which resulted in an extensive acute periostitis involving the teeth and jaw from the location of the second temporary molar of the left side, to the ramus of the jaw on the right side; all of the teeth between these points were loose, pus exuded from the gums at the necks of the teeth, and I feared extensive necrosis.

The treatment adopted was injections of $H_2$ $O_2$ beneath the gum at all points where pus was found to exude.

The conditions at the anterior part of the mouth began to improve at once, but opposite the first permanent molar at the lower margin of the jaw it was necessary a few days later to open an abscess which was about to point at that location.

The injections were kept up for two weeks; all discharges had then ceased, the teeth had become firm, and the patient was discharged.

Dr. Harlan has recently called attention to the use of $H_2 O_2$ in purulent conditions affecting the maxillary sinus, and I would suggest that it will be found equally valuable in the hands of the surgeon in nearly every variety of suppurative inflammation, especially in periostitis, necrosis, and deep-seated abscesses where there is difficulty in completely evacuating the purulent matter by the ordinary means.

